# The Competing Endogenous RNAs Regulatory Genes Network Mediates Leaf Shape Variation and Main Effector Gene Function in Mulberry Plant (*Morus alba*)

**DOI:** 10.3390/ijms242316860

**Published:** 2023-11-28

**Authors:** Jianbin Li, Lei Wang, Michael Ackah, Frank Kwarteng Amoako, Zijie Jiang, Yisu Shi, Haonan Li, Weiguo Zhao

**Affiliations:** 1 Jiangsu Key Laboratory of Sericulture Biology and Biotechnology, School of Biotechnology, Jiangsu University of Science and Technology, Zhenjiang 212100, China; 211111801108@stu.just.edu.cn (J.L.); 211211802105@stu.just.edu.cn (Z.J.); shiyisu1297@just.edu.cn (Y.S.); 221111802126@stu.just.edu.cn (H.L.); 2 Key Laboratory of Silkworm and Mulberry Genetic Improvement, Ministry of Agriculture and Rural Affairs, The Sericultural Research Institute, Chinese Academy of Agricultural Sciences, Zhenjiang 212100, China; 3 Institute of Plant Nutrition and Soil Science, Kiel University, Hermann-Rodewald-Straße 2, 24118 Kiel, Germany; stu225468@mail.uni-kiel.de

**Keywords:** *Morus alba*, leaf shape, competing endogenous RNAs (ceRNAs), GST-Pull down, miR156x/*Maspl3*/MSTRG.25812.1

## Abstract

Mulberry plants (*Morus alba*) have leaf shapes, ranging from unlobed to lobed, which are crucial for yield, growth, and adaptability, indicating their ability to adapt to their environment. Competing endogenous RNAs (ceRNAs) constitute a web of RNAs within the organism’s transcriptional regulatory system, including protein-coding genes (mRNAs), microRNAs (miRNAs), long non-coding RNAs (lncRNAs), circular RNAs (circRNAs), and others. In this study, samples for ceRNA sequencing were categorized into two groups: whole leaves and lobed leaves, each group with three replicates. In addition, we isolated, cloned, and characterized the precursor miRNA (miR156x) from the leaves of *M. alba*. miR156x precursor had a length of 107 base pairs and a minimum folding free energy of 50.27 kcal/mol. We constructed a pCAMBIA-35S-GUS-miR156x dual overexpression vector and established a transient transformation system for mulberry. At an optimal transformation solution (OD600 = 0.7), the GUS gene showed a higher expression in the leaves of transiently transformed mulberry with miR156x overexpression, four days after transformation, while the target genes of miR156x had decreased expression in the same leaves. Investigations into the transgenic mulberry plants uncovered various modifications to physio-chemical parameters including POD, SOD, PRO, MDA, soluble proteins and sugars, and chlorophyl content. miRNAs in the plants were found to act as negative regulators of gene expression in response to changes in leaf shape regulation, which was confirmed in vitro using dual-luciferase reporter assays. Subsequently, we cloned *Maspl3* in vitro and conducted GST-Pull down assays, obtaining multiple proteins that interacted with the *Maspl3* gene. This indicates that the miR156x/*Maspl3*/MSTRG.25812.1 regulatory module contributes to the differences in mulberry leaf shape.

## 1. Introduction

Mulberry (*Morus alba*) leaves consist of three parts: the petiole, leaf blade, and stipule. They are classified as complete leaves and serve as the primary assimilation organ and harvestable product of mulberry trees. The leaf shape of mulberry trees is an important agronomic trait, and variations in leaf shape indicate alterations in energy metabolism, environmental adaptation, and genetic variation [1]. Furthermore, mulberry trees are perennial woody plants, and their leaves are the fundamental source of food for the sericulture industry [2]. Mulberry leaves’ size and shape are significantly linked to their economic yield. Examining the growth, development, and morphogenesis of mulberry leaves, as well as the factors that influence leaf shape variation, can provide a strong basis for *M. alba* to be used as a model woody plant in the future. *M. alba* is a significant cultivated variety within the Moraceae family and *Morus* genus. It is primarily distributed in tropical, subtropical, and temperate regions and is widely found in China. *M. alba* has remarkable environmental resilience, being able to withstand stressful conditions. In its natural habitat, it can reach a height of shrubbery and can also live in harmony with other trees, forming a mixed forest with both trees and shrubs. This trait increases the biodiversity of the forest and helps to maintain its ecological balance [3,4]. It exhibits a range of leaf types, including entire, one-lobed, two-lobed, and, in some cases, up to six or seven-lobed leaves. Therefore, *M. alba* trees are the most suitable research material for studying the regulatory mechanisms of mulberry leaf shape.

In recent years, many research works have focused on the external morphological structures of mulberry leaves. Some researchers have also conducted in-depth analyses from a physiological perspective. Differences have been observed in the content of soluble proteins, superoxide dismutase (SOD) activity, peroxidase (POD) activity, malondialdehyde (MDA) content, proline (Pro) content, soluble sugar content, and chlorophyll content among the leaves [5,6]. However, there have been few reports on the identification of key genes in the regulatory networks responsible for leaf shape differences [7]. Therefore, utilizing modern molecular biology techniques as a core approach to analyze the crucial transcription factors (TFs) involved in mulberry leaf shape and understand related genes, proteins, and physicochemical is essential [7]. Ribonucleic acid (RNA) plays a crucial role in the transmission of genetic information between the genome and the proteome, serving as an essential intermediary in the central dogma of biology [8]. The messenger function of RNA has been widely accepted in the scientific community. Recent research works over the years have shown that small RNAs (sRNAs) have unique regulatory roles in the growth and development processes of both animals and plants. This demonstrates that RNA not only acts as a carrier of genetic information but also as a regulator of downstream target genes.

Competing endogenous RNA (ceRNA) does not refer to a new type of RNA molecule but rather to a complex network of RNAs within an organism’s transcriptional regulatory system [8,9]. This network includes protein-coding gene mRNAs, long non-coding RNAs (lncRNAs), pseudogenes, circular RNAs (circRNAs), miRNAs, and others [10,11,12,13]. Within these RNAs, there are regions that can be bound by miRNAs, known as microRNA response elements (MREs). By sharing MREs, the RNAs compete for the binding of the same miRNAs, and this interaction, mediated by miRNAs, influences and regulates the expression of target gene transcripts [14]. This collective regulation of gene transcription in the organism is a consequence of the interaction between these RNA molecules [15]. The ceRNA hypothesis complements the traditional miRNA → RNA paradigm, revealing a reverse regulatory mechanism of the RNA → miRNA interaction. In other words, ceRNAs compete for the same miRNAs through MREs, affecting the silencing of target genes caused by miRNAs [16]. The ceRNA hypothesis unveils a new mechanism of interaction between RNAs and has gained widespread attention in recent years, particularly in the fields of cancer research, pathology, growth, and development [17,18].

In plants, the temporary decrease in levels of miR156 controls nutritional development [19]. However, the specific process by which this decrease in expression is begun and maintained throughout leaf development is still not well understood. Research has shown that in *Arabidopsis* plants miR159 remains crucial for growth and development [20]. Loss of miR159 leads to an increase in miR156 levels throughout shoot development, delaying plant growth, while overexpression of miR159 slightly accelerates plant growth. The primary mechanism by which miR159 inhibits miR156 is by the targeting of *MYB33*, a transcription factor with an R2R3 MYB domain, by miR159 [20,21,22,23]. Despite significant downregulation of miR159, loss of *MYB33* still results in a premature flowering phenotype [24,25]. SPL3/4/5 are closely related members of the SQUAMOSA Promoter-Binding Protein-Like (*SPL*) transcription factor family in *Arabidopsis*, and their 3’UTRs contain a miR156-type target site [26,27,28]. Phenotypic investigation of *Arabidopsis* mutants containing *SPL*3/4/5 (both miR156-sensitive and miR156-insensitive variants) demonstrates that all three genes promote nutrient alterations and flowering and are substantially inhibited by miR156 [26,29,30]. The expression of miR156a has been shown to increase the duration of nutrient-related traits in seedlings while also shortening the flowering period. However, this phenotype is counteracted by the continual expression of the miR156-resistant form of *SPL3* [31]. As seedlings grow into the matured stage, miR156 levels steadily decrease, leading to a significant increase in *SPL3* mRNA abundance [32].

Study has it that miR156 in mulberry plant plays a major role in the transition to the juvenile phase, and overexpression of miR156 in transgenic mulberry trees significantly extends the juvenile phase [33]. Using degradation transcriptome sequencing and dual-luciferase reporter experiments, it was shown that a total of nine MnSPLs (Mulberry SQUAMOSA Promoter-Binding Protein-Like) could be cumulatively targeted by miR156, exerting direct regulation [33]. According to Li et.at. [33], results from dual-luciferase reporter assays indicated that six *MnSPLs* were able to recognize miR172 promoter sequences, subsequently activating their expression. This indicates that miR156 plays a role in mulberry trees by inhibiting the *MnSPL*/miR172 pathway. This research has illuminated the miR156/*SPL*s/miR172 regulatory pathway during mulberry tree development, thus bridging the gap in researchers’ understanding of the molecular mechanisms underlying the juvenile phase in perennial woody plants [33].

Due to the potential involvement of various types of RNA molecules in ceRNA effects, we employed a strategy of whole transcriptome sequencing in this study to detect all mRNA, lncRNA, circRNA, and small RNA molecules present in the samples, laying the foundation for analyzing their interactions. This research aims to uncover the ceRNA regulatory mechanisms underlying leaf shape differences in *Murus alba* and to reveal the impact of miR156x overexpression on physio-chemical traits in *M. alba* leaf morphogenesis. To ensure the reliability of ceRNA, we focused on the analysis of trustworthy RNA molecules. Based on the ceRNA sequencing results we obtained, we hypothesize in this study that miR156x can target and inhibit the expression of the *Maspl3* gene. This accession was validated by an in vitro experiment through dual-luciferase reporter assays. Furthermore, we conducted transient overexpression analysis of miR156x in plant cells, revealing that the overexpression of miR156x results in the upregulation of several genes, including *Maspl3*. Subsequently, we cloned *Maspl3* in vitro and performed GST-Pull down experiments, identifying multiple proteins that interacted with *Maspl3*. The transient transformation and transformation duration significantly induced changes in physiological and biochemical indicators such is SOD, POD, PRO, MDA, chlorophyll content, etc., involving the miR156x and target gene expression levels observed at day 4 of transformation. These results suggest that miR156x overexpression can increase the levels of certain enzymes and metabolites related to the regulation of leaf shape differences in mulberry trees. This, in turn, enhances their resistance to stress, further highlighting the molecular mechanisms involving miR156x and the target genes in *M. alba* the response to stress and adaptability. This indicates that the miR156x/*Maspl3* regulatory model contributes to the differences in mulberry leaf shape. The findings from this study support the role of miR156 as a regulator of *spl* gene expression in mulberry, shedding light on the complex regulatory mechanisms underlying mulberry leaf shape variation.

## 2. Results

### 2.1. Transcriptome Profile of ceRNAs and DEGs Analysis

**Figure 1:**
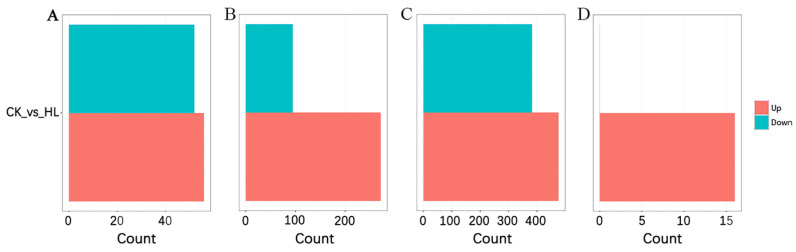
Analysis of ceRNA differential expression. (**A**) miRNA differential gene; (**B**) mRNA differential genes; (**C**) lncRNA differential gene; (**D**) circRNA differential gene.

The transcriptome profile on the sequencing information, mapping, etc., regarding the mRNAs, miRNAs, lncRNAs, and circRNAs are shown in the Supplementary File (Tables S1–S4). To identify the differentially expressed genes (DEGs) from the global transcriptome regulation underlying leaf shape differences in mulberry trees, mRNA and lncRNA selection criteria were set at FDR < 0.05 and |log2FC| > 1, while miRNA and circRNA criteria were set at *p* < 0.05 and |log2FC| > 1. From the results, the DEGs analysis revealed that among miRNAs, 56 were upregulated and 52 were downregulated. In the case of mRNAs, 2714 were upregulated and 947 were downregulated. Furthermore, for lncRNAs, 476 were upregulated and 383 were downregulated. Interestingly, there were 16 upregulated circRNAs with no downregulated counterparts (Figure 1). Volcan plot and heat maps showing the distribution pattern of the DEGs of the ceRNAs are shown in Figure S1.

### 2.2. Functional Enrichment Analysis

**Figure 2:**
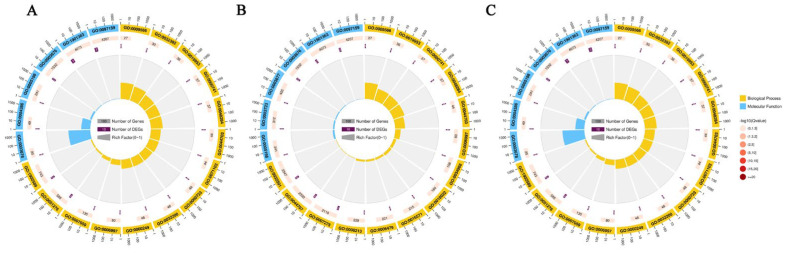
GO enrichment circle: (**A**) miRNA–mRNA enrichment circle; (**B**) lncRNA–mRNA enrichment circle; (**C**) circRNA–mRNA enrichment circle.

**Figure 3:**
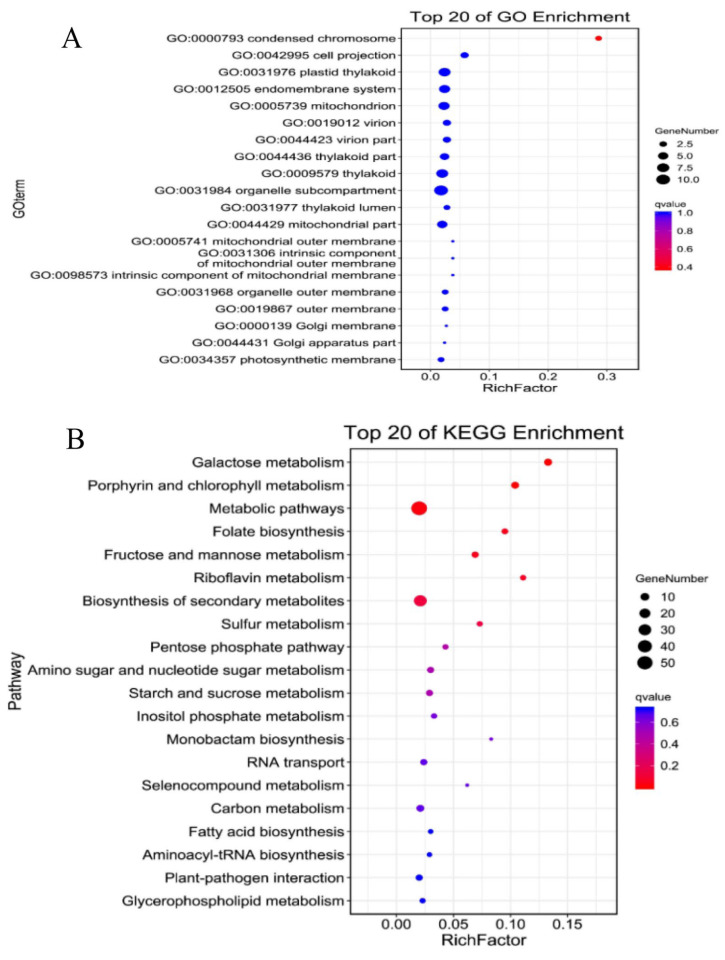
Enrichment analysis of the miRNAs target genes. (**A**) Top 20 GO enrichment. (**B**) Top 20 KEGG enrichment.

Functional analyses were carried on the DEGs targets of ceRNAs using both GO and KEGG databases. The results of the GO enrichment analysis indicate that miRNA-related genes were primarily involved in biological processes and molecular function, with the most enriched pathways being GO:1902589 and GO:1901363 (Figure 2A). In the case of lncRNA-mRNA-related genes, the genes were also implicated in a main involvement in biological processes and molecular function. Notable terms GO terms include GO:0032501and GO:0097159 (Figure 2B). Also, circRNA-mRNA-related genes were involved in similar GO functions and notable ones include GO:1902589 and GO:0097159 (Figure 3C). Analysis of the top 20 GO enrichments of the target genes shows that the genes were mainly involved in condensed chromosome, cell projection, and many others (Figure 3A). Also, analysis of the top 20 KEGG enrichments reveal that the genes were mainly enriched in galactose metabolism, porphyrin and chlorophyll metabolism, metabolic pathways pentose phosphate pathway, etc. (Figure 3B).

### 2.3. ceRNA Regulatory Network

We filtered and selected potential interacting ceRNAs through three aspects, including the targeting relationship and negative correlation of expression levels between miRNAs and candidate ceRNAs; the positive correlation of expression levels among candidate ceRNAs; and the enrichment degree of candidate ceRNAs binding to the same miRNA. As ceRNAs interact with each other through miRNAs, predicting target genes for differential miRNAs is the first step in the study of ceRNA regulatory networks. The results show that miRNA recruited several ceRNAs target including 64 mRNA, 9 lncRNA, and 4 circRNA (Table 1). We calculated the Spearman rank correlation coefficients for the target gene pairs between miRNAs and candidate ceRNAs, selecting target gene pairs with correlation coefficients less than or equal to −0.7 to obtain the differentially negatively correlated target miRNA–mRNA pairs (Table S5). This analysis uncovered that the miRNAs targeted seven *SPL* genes including miR156x targeting SQUAMOSA promoter-binding-like protein 3 (SPL3) (Table S5).

### 2.4. The DEG Validation by Real-Time RT-PCR Analysis

**Figure 4:**
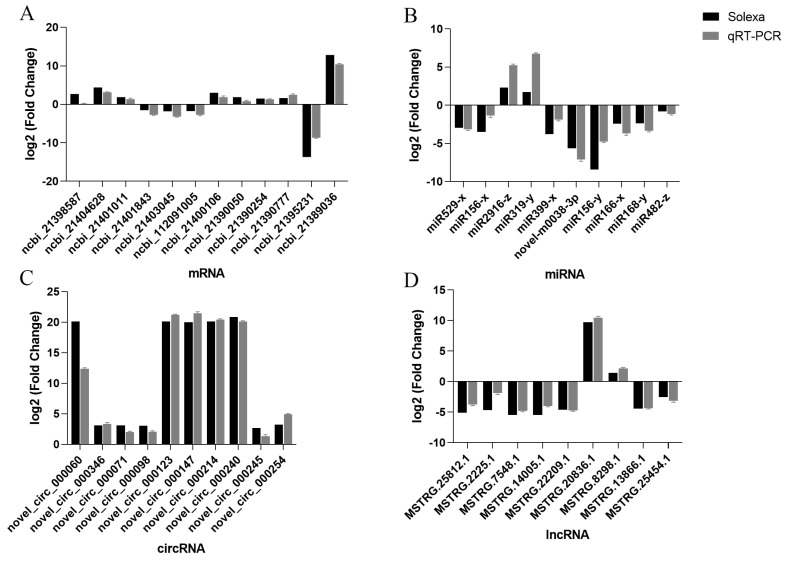
The verification of differentially expressed genes (DEGs) by qRT-PCR. (**A**) mRNAs. (**B**) miRNAs. (**C**) circRNAs. (**D**) lncRNAs.

Based on the ceRNA sequencing results, 12 pairs of mRNA, 10 pairs of miRNA, 9 pairs of lncRNA, and 10 pairs of circRNA target genes were randomly selected. Specific qRT-PCR primers were designed from the genes for validation, using β-actin as the internal reference gene (Tables S6–S9). The results demonstrated a consistent trend between the two methods, affirming the accuracy and reliability of the sequencing data (Figure 4).

### 2.5. Secondary Structure Analysis of miR156x

The mature sequence of miR156x is 5′-3′AACUGUCUUCUCUCUCUCGUG, with a length of 21 nucleotides. Using mfold (http://unafold.rna.albany.edu/?q=mfold, accessed on 10 September 2022) for pre-miR156x secondary structure prediction, based on the previously mentioned identification criteria, it was determined that the precursor sequence of miR156x is pre-miR156x. As shown in (Figure S2), miR156x exhibits the typical stem-loop structure characteristic of plant miRNAs.

### 2.6. miR156x Overexpression Analysis

**Figure 5:**
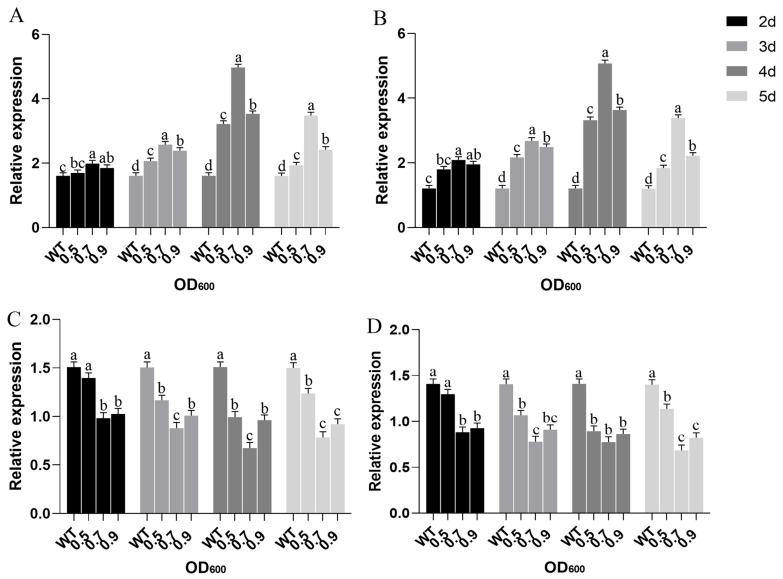
Relative expression level of different concentrations of transformation solution and different transformation days on genes. (**A**) GUS gene expression. (**B**) miR156x. (**C**) Target gene ncbi_21398587 expression after transient transformation of miR156x. (**D**) Target gene ncbi_21404628 expression after transient transformation of miR156x. Different letter symbols indicates statistical significance (Tukey’s HSD, *p* < 0.05).

After the identification of miR156x and its target *SPL* genes from the transcriptome data, we focused on exploring the role of miR156x and its target genes in regulating leaf morphology in mulberry plants. By constructing the miR156x-GUS fusion dual-element overexpression vector and establishing a transient transformation system in mulberry trees, we amplified the pre-miR156x extension sequence containing *EcoRI* and *HindIII* restriction enzyme sites. After double enzyme digestion, it was ligated into the pCAMBIA1305.1 expression vector. The resulting mulberry dual-element overexpression recombinant vector, pCAMBIA-35S-GUS-miR156x, comprises the CaMV35S promoter, NOS terminator, β-glucuronidase (GUS) reporter gene, and the pre-miR156x extension sequence (Figure S3). The results of the expression level of the GUS gene in transformed mulberry seedlings determined through qRT-PCR showed that there was a significant difference in GUS gene expression levels (*p* ≤ 0.01) when mulberry leaves were injected with recombinant *Agrobacterium* transformation solutions of different concentrations (Figure 5).

Furthermore, with increasing transformation solution concentration, the relative expression of GUS also increased. The highest relative expression of the GUS gene was observed when the transformation solution concentration was at OD600 = 0.7. However, as the transformation solution concentration continued to increase, the GUS gene expression level decreased, indicating that different transformation solution concentrations have a significant impact on the efficiency of transient transformation in mulberry. Using excessively high or low concentrations of transformation solution is not conducive to achieving transient transformation of the target gene. As shown in (Figure 5A), there were significant differences in the relative expression of the GUS gene with varying transformation times. In leaves treated for 4 days, the expression of GUS was the highest and significantly higher than other transformation durations (*p* ≤ 0.01). However, when leaves were treated for 5 days, the expression of the GUS gene decreased, indicating that transient transformation has a certain temporal effect.

The relative expression levels of miR156x at different transformation durations were assessed through qRT-PCR, and the results are depicted in (Figure 5B). After treatment with transformation solutions of various concentrations, the relative expression of miR156x significantly increased compared to the control group (*p* ≤ 0.05). The expression of miR156x attained the highest level at OD600 = 0.7 of the transformation solution concentration. This was a significantly higher OD600 of 0.5 and 0.9 (*p* ≤ 0.01) during the same period. On the 4th day of transformation, regardless of the transformation solution concentration, the expression of miR156x in the leaves was the highest (Figure 5A,B). At an OD600 of 0.5, the expression of miR156x was 2.74 times higher than that in the control group. Furthermore, at an OD600 of 0.7, the expression of miR156x increased approximately 4.20-fold compared to the control, and at an OD600 of 0.9, the expression of miR156x increased approximately 3.00-fold. However, on the 5th day of transient transformation, the expression of miR156x levels decreased. This further underscores the time-sensitive nature of *Agrobacterium*-mediated transient transformation.

Based on the high-throughput transcriptome sequencing results in mulberry trees, two target genes of miR156x, ncbi_21398587 (*SPL3*) encoding SQUAMOSA promoter-binding-like protein 3 and ncbi_21404628 (*SPL7*) SQUAMOSA promoter-binding-like protein 7, were selected. Through qRT-PCR, the impact of miR156x on the expression levels of these target genes was assessed, allowing for the determination of the regulatory role of miR156x in the control of mulberry leaf morphology changes. As depicted in (Figure 5C,D), following transient transformation of mulberry leaves with the recombinant *Agrobacterium* transformation solutions at concentrations of 0.5, 0.7, and 0.9, the expression levels of the two target genes of miR156x, *SPL3* and *SPL7*, decrease significantly from day 2 of treatment (*p* ≤ 0.05). At an OD600 of 0.7 and a transformation duration of 4 days, the expression levels of the two target genes were at their lowest compared to the control group. Among them, *SPL3* exhibited the most significant expression change, with a 2.24-fold decrease compared to the control, while *SPL7* showed a 1.82-fold decrease. Conversely, at the same transformation concentration and duration, the expression of miR156x increased fourfold compared to the control, indicating that miR156x exerts an inhibitory effect on target gene expression, thereby regulating the growth and development processes, including leaf morphology changes, in mulberry trees.

### 2.7. The Impact of miR156x Overexpression in Mulberry Plant on Physiological and Biochemical Indicators Related to Leaf Morphology Regulation

**Figure 6:**
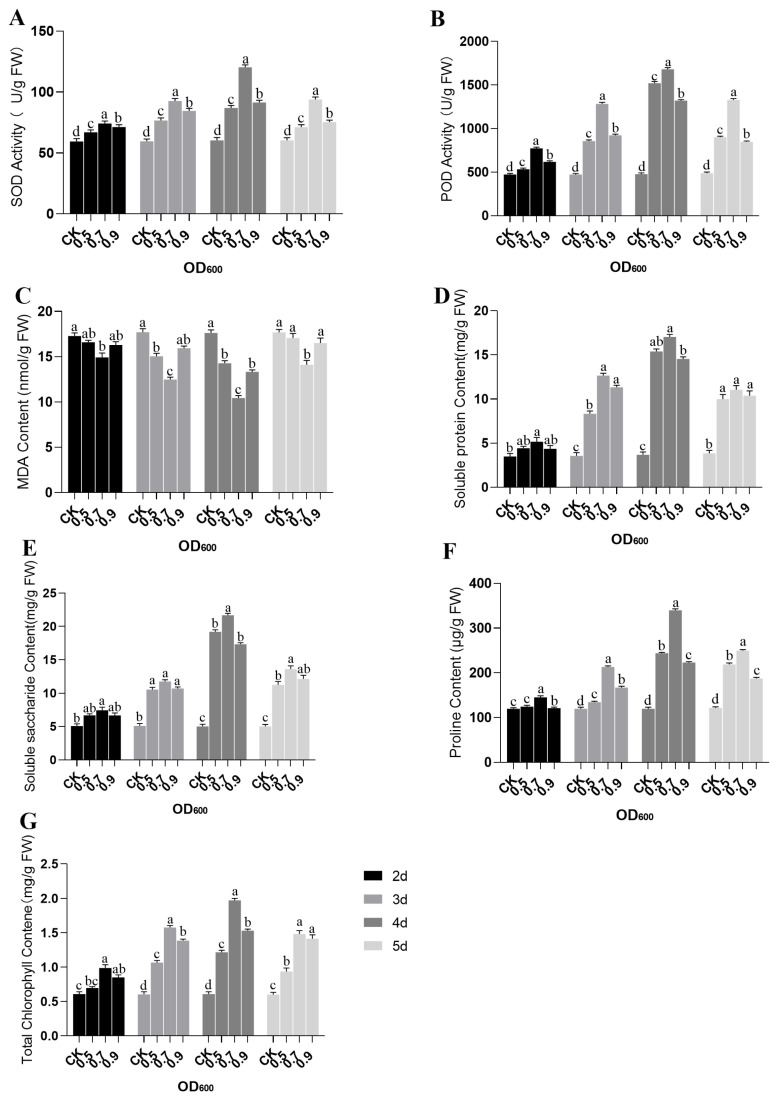
Effects of miR156x overexpression on physiological and biochemical indexes related to leaf shape regulation. (**A**) Variation in soluble protein content after instantaneous conversion of miR156x. (**B**) Variation in SOD activity after instantaneous conversion of miR156x. (**C**) Variation in POD activity after instantaneous conversion of miR156x. (**D**) Variation in MDA content after instantaneous conversion of miR156x. (**E**) Variation in proline content after instantaneous conversion of miR156x. (**F**) Changes in soluble sugar content after transient transformation of miR156x. (**G**) Variation in chlorophyll content after instantaneous conversion of miR156x. Different letter symbols indicates statistical significance (Tukey’s HSD, *p* < 0.05).

Important physiological and biochemical indicators related to plant stress resistance were measured to investigate the physiological differences between transiently transformed miR156x-overexpressing mulberry seedlings and control mulberry seedlings. These results contribute to the study on the role of miR156x in the variation in mulberry leaf morphology. From the analysis, the results reveal that in mulberry seedlings, when miR156x overexpression was induced with transformation solution concentrations with an OD600 of 0.5, 0.7, and 0.9, the activity of SOD significantly increased from days 2 to 5 (*p* ≤ 0.01) (Figure 6A). Under the same transformation solution concentration, SOD activity continued to increase with the duration of transformation, reaching its highest level at day 4 of transformation, suggesting that overexpression of miR156x has a notable enhancing effect on SOD activity, thereby playing a role in mulberry leaf morphology changes.

Overexpression of miR156x in mulberry leaves was demonstrated to significantly increase POD activity in the leaves, as illustrated in Figure 6B. Transient overexpression of miR156x with OD600 concentrations of 0.5, 0.7, and 0.9 in mulberry seedlings resulted in a highly significant increase in leaf POD activity from days 2 to 5 (*p* ≤ 0.01). The experimental results demonstrate that miR156x can significantly increase the activity of POD in plants, enhancing their antioxidant capacity. This suggests that miR156x plays a positive role in mulberry leaf morphology changes and growth and development processes.

Transient overexpression of miR156x in mulberry trees did not cause a significant change in MDA content at different transformation solution concentrations for the first two days (Figure 6C). However, from day three onwards, the levels of MDA were significantly higher than the control (*p* < 0.01). Under the same transformation solution concentration, MDA levels decreased with the increasing duration of transformation, reaching their lowest point on the 4th day. At an OD600 of 0.7 and a transformation duration of 4 days, the decrease in MDA was the most drastic, whereas a lower OD600 concentration of 0.5 led to the smallest decrease in MDA. This suggests that overexpression of miR156x can reduce the production of MDA in mulberry tissues, thus mitigating lipid membrane oxidation and altering the leaf morphology of mulberry trees.

Data presented in Figure 6D demonstrate that when miR156x was overexpressed in mulberry seedlings, the content of soluble proteins in mulberry leaves rose significantly (*p* ≤ 0.05). All transformation solution concentrations resulted in a highly significant increase (*p* ≤ 0.01) in soluble proteins, with the highest levels being observed on the fourth day of transformation under conditions with an OD600 of 0.7. It was also observed that, when compared to other transformation solutions, an OD600 of 0.7 produced a higher amount of soluble protein content during the same transformation duration, implying that an OD600 of 0.7 is likely the optimal concentration for miR156x transient transformation in mulberry trees.

Analysis of mulberry seedlings revealed that overexpression of miR156x, induced by transformation solution concentrations of OD600 at 0.5, 0.7, and 0.9, significantly altered the content of soluble sugars in mulberry trees from the first two to five days of transformation (Figure 6E). Notably, the content of soluble sugars was significantly different from the control group after four days of transformation (*p* ≤ 0.01), with the highest level being observed on the fourth day. At a transformation duration of 4 days, the highest concentration of soluble sugar was observed in the leaves when the OD600 concentration was 0.7, followed by 0.9, compared to 0.5. This suggests that transient overexpression of miR156x in mulberry trees can regulate leaf morphology by increasing the accumulation of soluble sugars.

Proline (PRO) is a common component of various crops, and its content can be used as a physical and chemical indicator for varietal breeding. As illustrated in Figure 6F, when miR156x was overexpressed at transformation solution concentrations of 0.5, 0.7, and 0.9, there was no significant difference in Pro content in mulberry plants during the first two days. However, from the third day onwards, there was a highly significant difference in Pro content compared to the control (*p* ≤ 0.01). It is interesting to note that Pro content increased to its highest level on the fourth day. At an OD600 concentration of 0.7 and a transformation duration of 4 days, Pro content exhibited the highest increase, followed by 0.9 and 0.5 concentrations of the transformation solution, indicating the influence of transient overexpression of miR156x on Pro content in mulberry trees, thus regulating mulberry leaf morphology.

Chlorophyll, which is found in abundance in plant tissues, is closely associated with photosynthesis and nutrition. It is a reliable measure of a plant’s ability to absorb nutrients and can be used to gauge a plant’s growth and development. Results from the experiment revealed that mulberry seedlings induced with transformation solutions with OD600 concentrations of 0.5, 0.7, and 0.9, overexpressing miR156x from the second day of transformation, had significantly (*p* < 0.01) higher total chlorophyll content in their leaves compared to control leaves (Figure 6G). The total chlorophyll content in mulberry plants was most notably augmented when the OD600 was 0.7 and at 4 days of transformation, whereas the least increase was seen when OD600 was 0.5. This suggests that the total chlorophyll content in mulberry trees is sensitive to the overexpression of miR156x.

### 2.8. Cloning and Verification of the MaSPL3 Gene in Mulberry

**Figure 7:**
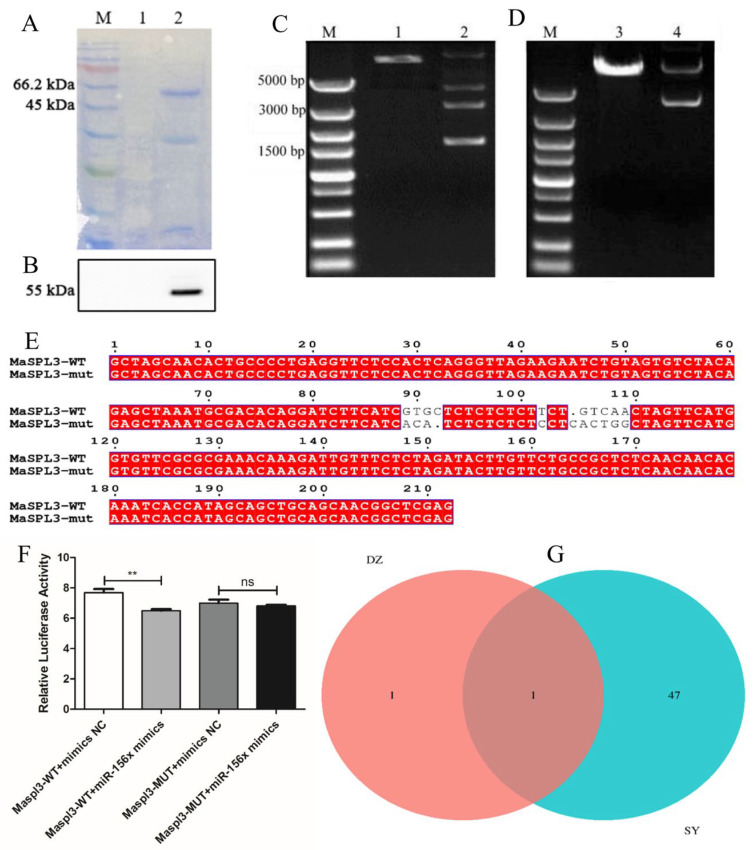
Cloning and verification of the *MaSPL3* and miR156x genes in mulberry. (**A**) Induced expression of recombinant His-MaSPL3 protein in *Escherichia coli* BL21 cells. (**B**) Western blot detection of recombinant His-*MaSPL3* protein; M: Marker, 1. before induction, 2. after induction. (**C**) pmirGLO-*MaSPL3*-WT plasmid digestion result. (**D**) pmirGLO-MaSPL3-MUT 1, 3 plasmid digestion result. Nhe Ⅰ enzyme digestion plasmid, 2, 4 plasmid. (**E**) *MaSPL3* mutation site. (**F**) *MaSPL3* and miR156x double luciferase. (**G**) Venn diagram of differential proteins after SDS-PAGE analysis. ** sign indicated statistical significance level at t-text (*p* ≤ 0.05). ns mean nonsignificant at t-text (*p* < 0.05). Red color is the MaSPL3 gene sequence, and the grey letters are the mutated part of the sequence.

The specific primers *MaSPL3*-F1/R1, designed based on the homologous conserved sequences found on NCBI, were used for PCR amplification (Table S10). Sequence alignment against the target product sequence using NCBI confirmed that the cloned sequence is correct and shares high homology with *SPL3* genes from Chinese mulberry and other species. The obtained *MaSPL3* gene (GenBank No: XM_024169654) has a cDNA sequence encoding 500 amino acids (Figure S4), with a protein molecular weight of 55.04 kDa and an isoelectric point of 8.83. This gene was named *MaSPL3* and represents the first cloning of an *SPL3* gene in *M. alba*. To gain further insight into the protein structure of *MaSPL3*, its tertiary structure was predicted using Swiss-Model, as shown in (Figure S5). The *M. alba SPL3* (*MaSPL3*) gene was cloned, and its expression pattern was analyzed. The target protein was obtained through prokaryotic expression; (Figure S6) illustrates the schematic representation of the prokaryotic expression vector pET-20b(+)-*MaSPL3* construction for *M. alba*. SDS-PAGE analysis results revealed that the recombinant protein of *MaSPL3* was produced in the form of inclusion bodies, with a predicted molecular weight of 55 kDa, consistent with the observed band on the SDS-PAGE gel (Figure 7A). After SDS-PAGE, the induced recombinant protein was transferred to a nitrocellulose membrane and subsequently analyzed by Western blot. This analysis, using mouse anti-His-tag primary antibody and HRP-conjugated goat anti-mouse secondary antibody, confirmed that the induced protein was *MaSPL3* (Figure 7B).

### 2.9. Dual Luciferase Validation of the miR156x and Maspl3 Targeting Relationship

**Figure 8:**
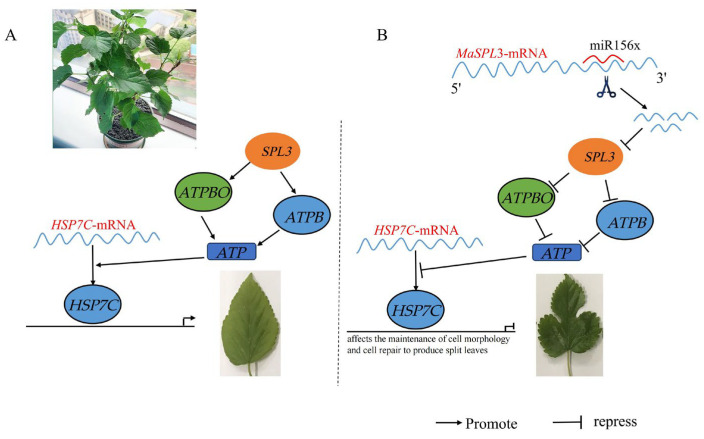
Schematic representation of the proposed molecular mechanism involving miR156x and *MaSPL3* genes in mulberry. (**A**) *Morus alba* plant. (**B**) Putative molecular mechanism of miR156x and *MaSPL3* genes. SPL (SQUAMOSA promoter-binding protein-like), HSP (Heat shock proteins) ATPs (ATP synthase; ATPB (sp|P19366|ATPB_ARATH) encodes the β subunit; ATPBO (sp|Q9C5A9|ATPBO_ARATH) encodes the δ subunit). Scissors indicated miRNA cleavage; arrows and bar sign indicated the genes promote and repress leaves morphology, respectively.

Plasmids pmirGLO-MaSPL3-WT and pmirGLO-MaSPL3-MUT were constructed, with the mutation site as shown in (Figure 7E). The concentration of pmirGLO-*MaSPL3*-WT was 0.1427 μg/μL, while that of pmirGLO-*MaSPL3*-MUT was 0.1518 μg/μL. Plasmid digestion is illustrated in (Figure 7C,D). Following successful sequencing, pmirGLO-*MaSPL3*-WT was transfected into 293T cells in combination with miR-156x mimics. The results indicate that compared to the pmirGLO-*MaSPL3*-WT+mimics NC group, the fluorescence intensity was significantly reduced. Similarly, when pmirGLO-*MaSPL3*-MUT was transfected into 293T cells with miR-156x mimics, there was no notable difference in fluorescence intensity compared to the pmirGLO-*MaSPL3*-MUT+mimics NC group (Figure 7F). This further confirms the presence of a targeted interaction between *MaSPL3* and miR156x, where miR156x is able to inhibit the expression of *MaSPL3* (Figure 8).

### 2.10. GST-Pull Down Analysis of the Interacting Proteins of MaSPL3 in Mulberry Plants

By using the Proteinpilot software (v 4.5) connected to the AB SCIEX Triple TOF™ 5600 plus mass spectrometer (Redwood City, CA, USA), the raw data obtained from LC-MS/MS were analyzed and compared between the experimental group (SY) and control group (DZ). With a confidence level of 95 and a minimum of one unique peptide, the SY group produced 7096 secondary mass spectra while the DZ group generated 2487 (Table 2). The number of interpreted secondary mass spectra was 915 for the experimental group and 574 for the control group.

In this experiment, two samples were examined, and it was found that the identified proteins not only vary in quantity, but also some proteins were exclusive to certain samples while others were shared between the samples. A Venn diagram (Figure 7G) was created to display the protein sets with variations between the DZ and SY samples, which totaled 49 proteins. Out of the proteins identified, one was present in both samples, while DZ and SY had one and forty-seven proteins exclusively, respectively. Detailed analysis of the top-ranked proteins in each sample, with a confidence level of 95% and a minimum of one unique peptide, is presented in Table 3, which provide information about the proteins.

## 3. Discussion

miRNAs play a pivotal role in regulating numerous fundamental cellular processes [34]. In plants, many biological functions rely on the normal functioning of miRNAs, including the maintenance of chromosomal states, gene expression regulation, promotion of mRNA stability, control of protein translation, resistance to stress, and defense responses against pathogens [35]. Studies on several relatively conserved miRNAs have revealed that they typically act as negative regulators by targeting downstream mRNA of target genes, often transcription factors [36]. For instance, miR156 and miR172 exhibit opposing regulatory roles during the transition from vegetative to reproductive growth in plants. MiR156 exerts inhibitory effects on downstream transcription factors, such as SQUAMOSA promoter-binding-like protein and APETALA2 (*AP2*), while miR172 activates these two genes’ transcription by inhibiting its downstream target genes [37]. The SPL protein family represents a plant-specific transcription factor family, and mature miR156 can complementarily bind to target sites in *SPL* mRNA, leading to the cleavage of *SPL* transcripts [38]. The regulation of vegetative development in plants is subject to temporal control, which is characterized by a temporary decrease in miR156 expression. Despite this, the mechanisms underlying the initiation and maintenance of this reduced expression during leaf development remain unclear. Studies have indicated that increasing miR156 expression levels throughout the entire shoot development process in *Arabidopsis* can slow down plant growth, whereas reducing miR156 expression slightly accelerates plant growth [39].

Mulberry trees possess a long growth cycle, a complex genetic background, and a lack of a stable regeneration and genetic transformation system, making the study of miRNA function in them challenging [40]. *Agrobacterium*-mediated transient expression technology is a technique for rapidly introducing foreign genes into plant recipient cells, allowing for the transient high-level expression of the target gene. Compared to stable genetic expression, this technique offers advantages such as ease of operation, speed, and high feasibility, and it is widely adopted [41]. In this study, a dual-element overexpression vector for mulberry miR156x was constructed, and a transient transformation system for mulberry trees was established. Several aspects were explored, including the effects of different *Agrobacterium* culture concentrations and different transformation durations on the efficiency of transient transformation in mulberry, as well as the changes in GUS gene and miR156x expression levels. The study also analyzed the regulatory function of miR156x in mulberry leaf shape variation by examining its effects on target gene expression levels and mulberry tree physiological and biochemical indicators.

A mulberry miR156x dual-element overexpression vector, pCAMBIA-35S-GUS-miR156x, was constructed, containing the CaMV35S promoter, NOS terminator, β-glucuronidase (GUS) reporter gene, and pre-miR156x extension sequence. Comparative analysis after transient overexpression of miR156x revealed that when the OD600 concentration of the transformation solution was 0.7, and at 4 days of transformation duration, the expression levels of the GUS gene and miR156x in the leaves were significantly higher compared to control leaves. This indicates that the appropriate concentration of the transformation solution has a significant impact on transient transformation efficiency, and *Agrobacterium*-mediated transient transformation has a certain degree of temporal specificity. The experiment found that when mulberry leaves were transiently transformed with recombinant *Agrobacterium* transformation solutions with OD600 concentrations of 0.5, 0.7, and 0.9, the expression levels of two HD-Zip target genes and one JMJD6 target gene of miR156x significantly decreased from 2 days after transformation. When the OD600 concentration of the transformation solution was 0.7 and the transformation duration was 4 days, the expression levels of these three target genes were the most significantly reduced compared to control leaves. Under the same treatment, the expression of miR156x increased by more than 4 times compared to the control. This suggests that miR156x can significantly counter-regulate the expression levels of its target genes and assist mulberry trees in regulating leaf shape changes.

After transient overexpression of miR156x in mulberry trees using different concentrations of transformation solutions, several physiological and biochemical indicators showed varying degrees of increase from 2 days after transformation when compared to the control treatment. These indicators include soluble protein content, SOD (superoxide dismutase) activity, POD (peroxidase) activity, Pro (proline) content, and chlorophyll content. The most significant increase in these physiological and biochemical indicators occurred when the *Agrobacterium* culture concentration was OD600 0.7, and the transformation duration was 4 days. SOD is widely distributed in plant tissues and catalyzes the dismutation of superoxide anions (O_2_^−^) into hydrogen peroxide (H_2_O_2_) and oxygen (O_2_). It effectively prevents and controls the increase in oxygen free radicals, which can damage cells. Additionally, SOD plays a role in the timely repair of injured cells as a result of reactive oxygen species (ROS) accumulation. Concurrently, MDA (malondialdehyde) content showed the most significant decrease under these conditions. Following the completion of miR156x overexpression, both the relevant target genes and physiological and biochemical indicators in the leaves underwent changes. The changes in the trend of culture concentration levels and miR156x target gene expression were consistent with the changes in physiological and biochemical indicators related to leaf shape differences. During the transient transformation process, due to differences in transformation duration, there were variations in the results, with the most significant changes in physiological and biochemical indicators and target gene expression levels observed at day 4 of transformation. These results suggest that miR156x overexpression can increase the levels of certain enzymes and metabolites related to the regulation of leaf shape differences in mulberry trees. This, in turn, enhances their resistance to stress, further highlighting the positive role of miR156x in the response of mulberry trees to stress and adversity.

SQUAMOSA promoter-binding protein-like (SPL) protein family is a plant-specific transcription factor family [42]. Through whole transcriptome sequencing, it has been established that the *MaSPL3*-miR156x regulatory pathway plays a significant role in shaping the leaves of mulberry trees. *SPL3*, in particular, plays a crucial regulatory role in plant growth [26,43,44]. Within *SPL* 3′UTR (3′ untranslated region), there is a target site for miR156 [42]. The phenotypic characteristics of mulberry trees, such as leaf shape, are strongly influenced by *SPL3*, which is strongly inhibited by miR156 [45]. Constitutive expression of miR156a prolongs the expression of juvenile leaf traits and leads to differences in leaf morphology. This phenotype is largely corrected by constitutive expression of miR156-insensitive forms of SPL3 [46]. In this study, a new *SPL3* gene was cloned from the *M. alba*. The full-length cDNA sequence of *MaSPL3* was found to be 1,503 bp in length, encoding a protein with 500 amino acid residues, a molecular mass of 55.04 kDa, and an isoelectric point of 8.83. Conservation domain analysis showed that the MaSPL3 protein domain is highly conserved and contains distinct protein kinase domains, including the ATP-binding domain and serine/threonine protein kinase activation domain, belonging to the plant *GSK3*/shaggy protein kinase family. Glycogen synthase kinase (*GSK3*) belongs to the serine/threonine protein kinase family and plays a crucial role in plant organ development, hormone signaling, and responses to biotic and abiotic stresses.

Luciferase is a collective term for enzymes found in nature that can produce bioluminescence [47]. It is a relatively common enzyme, with the most representative forms being derived from fireflies (F-Luciferase) and Renilla (R-Luciferase). These two types of luciferase enzymes catalyze substrates into oxyluciferin, which exhibits strong luminescent capability [48]. Luciferase enzymes efficiently oxidize luciferin into oxyluciferin and generate bioluminescence in the presence of Mg^2+^ and O_2_ [49]. Traditionally, this technology has been used to measure promoter activity and study the relationship between target gene promoters and transcription regulatory factors. It can also be used to investigate the impact of miRNAs on downstream target genes. Dual-luciferase reporter gene analysis is a system that combines firefly luciferase and Renilla luciferase assays [50]. In gene expression analysis, a common approach is to simultaneously transfect two reporter genes and modulate the promoter linked to the reporter gene.

Changes in reporter gene expression are related to changes in the transcriptional activity of the modulated promoter. The other reporter gene, which constitutes the promoter’s transcriptional activity, serves as an internal control to ensure that the experiment is not influenced by environmental factors [51]. To study the strength of promoters and the role of transcription factors or the regulatory effects of miRNAs on target genes or lncRNAs, transcription regulatory elements or 5′ promoter regions of the target gene can be cloned upstream of the Firefly luciferase gene, or 3′-UTR regions or lncRNA binding sequences can be cloned downstream of the Firefly luciferase gene [52]. In this study, when wild-type miR156x was introduced as mimics, a significant reduction in fluorescence intensity was observed, indicating that the presence of miR156x inhibited a specific fluorescent signal. However, after introducing a base mutation in miR156x, there was no significant change in fluorescence intensity compared to the control group. These results suggest the presence of a target interaction between *MaSPL3* and miR156x. Specifically, miR156x exerts its regulatory effect by targeting the mRNA of *MaSPL3*, leading to the suppression of *MaSPL3* expression (Figure 7 and Figure 8).

Proteomic identification is a central technique in proteomics research, and currently, it primarily relies on mass spectrometry for protein identification [53]. Mass spectrometry can be categorized into two main types: MALDI-TOF and LC-MS/MS. Among these, LC-MS/MS stands out as the most commonly used method due to its higher sensitivity and its reliability [54]. The workflow typically involves different types of samples, including protein–protein interaction samples, gel strips and spots, protein solutions, and others. After protein extraction and enzymatic digestion, which can be optimized using in-gel or in-solution methods, LC-MS/MS is employed to analyze the peak patterns and charges of protein fragments. This process significantly enhances the accuracy of mass spectrometry identification and peptide coverage [55]. GST pull-down experiments utilize recombinant technology to express probe proteins fused with GST (Glutathione S-transferase). The fusion protein, with GST, can bind to immobilized GTH (Glutathione) on a solid phase support [56]. When proteins that interact with this fusion protein pass through a chromatography column, they can be adsorbed by the solid-phase complex, leading to their separation [57]. This experiment first establishes a GST pull-down system, then expresses fusion proteins with GST tags using a prokaryotic expression system, purifies the proteins to high purity using a GST affinity purification column, and finally detects protein interactions using the GST affinity purification column [58]. A total of 49 proteins were co-identified, with two samples overlapping in the identification of one protein. DZ and SY identified one and 47 unique proteins, respectively. Many of these proteins belong to the *SPL* family, indicating their involvement in mulberry leaf morphogenesis. The study of other genes within this family holds significant importance in understanding the role of *SPL* family proteins in mulberry tree leaf development.

## 4. Materials and Methods

### 4.1. Plant Material, Growth Conditions, and Treatments

*M. alba* was collected from the Jiangsu University of Science and Technology National Mulberry Germplasm Repository in Zhenjiang, China. Random leaf samples from both the entire leaves and the aberrant plants’ leaves were collected from the germplasm repository and rapidly frozen in liquid nitrogen for subsequent experiments. In addition, *M. alba* seedlings were transplanted into containers containing a vermiculite and soil mixture (pH 7.0). When the seedlings reached a height of approximately 20 cm, they were randomly divided into three groups. Furthermore, *Agrobacterium tumefaciens* (LBA4404) injection served as the control (CK) and *Agrobacterium tumefaciens* (LBA4404), carrying the miR156x dual-overexpression vector with OD600 values of 0.5, 0.7, and 0.9, were used as experimental groups. Treated samples were collected at 2, 3, 4, and 5 days after treatment.

### 4.2. RNA Isolation, Library Construction, and Sequencing

Following the manufacturer’s protocol, RNAiso Plus reagent (Takara, Beijing, China) was used to extract total RNA from fresh leaf samples. The Nanodrop UV-Vis spectrophotometer (Metash, Shanghai, China) was used to determine the concentration and purity of the total RNA, and its quality was checked with 1.0% agarose gel electrophoresis. The total RNA extracted was subjected to ribosomal RNA (rRNA) depletion to prepare a strand-specific lncRNA, miRNA, and circRNA library. The Agilent 2100 Bioanalyzer (Agilent Technologies, California, CA, USA) and the ABI StepOnePlus Real-Time PCR System (Thermo, Waltham, MA, USA) were utilized to evaluate the quality and yield of the constructed library. To obtain cDNAs, reverse transcription of the extracted total RNA was carried out using the PrimeScript™ RT Kit (Takara, Beijing, China), following the manufacturer’s instructions. Subsequently, high-throughput sequencing was performed on the Illumina Hiseq 2500 (Illumina, San Diego, CA, USA) platform using the PE150 (paired-end 150 bp) sequencing mode.

### 4.3. Sequence Data Analysis and Assembly

After sequencing, a rigorous data filtering process was applied to eliminate low-quality and irrelevant information. Thereafter, the obtained data were compared with the *M. notabilis* reference genome. Unmapped Reads, representing sequences that do not align with the reference genome, were then extracted and further refined by trimming both ends, typically 20 base pairs each, and, finally, the complete Anchors Reads were obtained and the clean reads were compared with the genome again. The transcriptome was reconstructed using stringtie, facilitating the assembly of transcripts. Additionally, the coding capacity of these transcripts was predicted using CPC software v3.2.0.

### 4.4. ceRNAs Network Construction and Functional Enrichment Analysis

By utilizing the edgeR software package v3.2.4, we identified the differentially expressed transcripts among various samples or groups. We defined mRNAs and lncRNAs with the criteria of fold changes ≥ 1 and a false discovery rate (FDR) < 0.05 as significant differentially expressed genes (DEGs), while miRNAs with fold changes ≥ 1 and a *p*-value < 0.05 were considered important DEGs. For plant samples, we employed software to predict miRNA target genes. To construct a ceRNA network according to the ceRNA theory, we obtained the sequences and family information of miRNAs from the TargetScan website (https://www.targetscan.org/vert_80/ accessed on 5 October 2022)and then evaluated the expression correlation between mRNA–miRNA or lncRNA–miRNA pairs utilizing the Spearman Rank correlation coefficient (SCC). Pairs with a SCC less than −0.7 were identified as co-expressed negative lncRNA–miRNA or mRNA–miRNA pairs, wherein both mRNA and lncRNA were target genes of miRNA and all RNAs were differentially expressed. To evaluate the expression correlation between lncRNA–mRNA pairs, the Pearson correlation coefficient (PCC) was used, and pairs with a PCC greater than 0.9 were chosen as co-expressed lncRNA–mRNA pairs. In these pairs, both mRNA and lncRNA were targeted by the same miRNA and co-negatively expressed. We used the hypergeometric cumulative distribution function to test whether the shared miRNA sponge between two genes was statistically significant. Thus, we only selected gene pairs with a *p*-value less than 0.05.

### 4.5. Functional Enrichment Analysis

Gene Ontology (GO) biological process term and Kyoto Encyclopaedia of Genes and Genomes (KEGG) pathway analyses were performed on the mRNAs within the ceRNA network in order to evaluate the functional enrichment of the DEGs. In the process, ceRNAs associated with biological functions were filtered out. The mapping of all ceRNAs to GO terms was performed using the Gene Ontology database (http://www.geneontology.org/; accessed on 10 October 2022). Gene counts for each term were calculated, and significantly enriched GO terms in ceRNAs compared to the genomic background were identified using hypergeometric testing [59]. Pathway analysis of the genes was analyzed based on the KEGG database (http://www.kegg.jp/kegg; accessed on 10 October 2022) [60]. Pathway enrichment analysis determined which metabolic pathways or signaling pathways are significantly enriched in ceRNAs compared to the genomic background.

### 4.6. Quantitative Real-Time PCR (qRT-PCR) Verification of DEGs

To validate the accuracy of the sequencing results, qRT-PCR technology was employed to randomly select 12 mRNAs, 10 miRNAs, 9 lncRNAs, and 10 circRNAs for validation. Primers’ information is available in Tables S3–S6. The specific procedures followed Li et al. [33], with each sample having three biological replicates. U6 was used as the reference gene for miRNA, while 18S rRNA served as the reference gene for others. Data analysis was performed using the 2^−ΔΔCt^ method [61], and the results were transformed to log_2_ values for the analysis.

### 4.7. PCR Amplification and Prokaryotic Expression Vector Construction

The amplification of the target *MaSPL3* gene was performed by using a PCR amplification kit (Monad Biotech Co., Suzhou, China) according to the manufacturer’s instructions. Primers with NdeI and XhoI restriction enzyme sites were designed using Primer 5.0, and their sequences were as follows: *MaSPL3*-F: GCCATATGATGGAGTGGAATTCAA and *MaSPL3*-R: CCGCTCGAGCTTGAAGTGATGGGA. The reaction mixture included 4 μL of cDNA, 2 μL of *MaSPL3*-F (10 μM), 2 μL of MaSPL3-R (10 μM), 25 μL of 2× Taq mix, and ddH2O was added to reach a final volume of 50 μL. The PCR amplification was conducted using the following cycling conditions: 1 min at 95 °C for denaturation, followed by 35 cycles of denaturation at 95 °C for 15 s, annealing at 60 °C for 20 s, and extension at 72 °C for 1 min, with a final extension at 72 °C for 2 min. The PCR-amplified target fragment was then ligated into the pET-20b vector with NdeI and XhoI sites using a double enzyme digestion method, following the manufacturer’s instructions. The ligated plasmid was transformed into *Escherichia coli DH5α* competent cells through heat shock. Positive colonies were selected and confirmed by both PCR and sequencing. Plasmids that were verified by sequencing were transformed into *Escherichia coli* BL21 competent cells for prokaryotic expression through heat shock.

### 4.8. Sequence Analysis of the miR156x Precursor

In the analysis of the precursor sequence of miR156x in *M. alba*, we initiated a search for its precursor sequence within the mulberry genome database, accessible at http://morus.swu.edu.cn/morusdb/; accessed on 5 October 2022). Subsequently, upon identification of the precursor sequence, it was extended by 200 base pairs at both the 5′ and 3′ ends to encompass the complete pre-miR156x sequence. To predict the secondary structure of this pre-miR156x sequence, we utilized the mfold online software (http://mfold.rna.albany.edu/?q=mfold/download-mfold; accessed on 7 October 2022), adhering to specific criteria such as a folding free energy below −18 kcal/mol, a folding free energy value of ≥0.85, a limit of ≤3 asymmetrically bulging base pairs, and a maximum of ≤4 mismatched base pairs. Additionally, to enhance the confidence in the identified pre-miR156x sequence and to predict its secondary stem-loop structure, a homology analysis with closely related species within the Rosales order was performed.

### 4.9. Analysis of miR156x Overexpression Patterns

We constructed the miR156x-GUS bicistronic overexpression vector: Overlap PCR was used to amplify pCAMBIA1305.1 and the CaMV 35S promoter (enhanced) from the mulberry genome (CZF1:GATCGAATTCTCTTCGTCAACATGGTGGAGCACG,; CZR1: ACTAGGCTTTGCCGAAGCAGTGTTCTCTCCAAATGAAATG); pre-miR156x (CZF2: CATTTCATTTGGAGAGAACAGTATGGGCGAACGACGGGAA); CZR2: TGCCAAATGTTTGAACGATCTCAACCAATCTGTAGTTGAT); and NOS terminator (CZF3: ATCAACTACAGATTGGTTGAGATCGTTCAAACATTTGGCA), CZR3: GATCAAGCT TATCTAGTAACATAGATGACACCGCGC). CZF1 and CZR3 contained *EcoRI* and *HindIII* restriction enzyme sites. The fused three fragments were ligated into the pCAMBIA1305.1 vector (Takara, Beijing, China) using T4 ligase and confirmed by sequencing. Furthermore, the correctly sequenced plasmids were transformed into *Agrobacterium tumefaciens* strain LBA4404 using the freeze–thaw method. *Agrobacterium* strains with positive results were suspended in an infection buffer composed of 10 mM MgCl2, 10 mM MES, pH 5.7, and 150 mM acetosyringone (AS), and then injected into mulberry leaves under pressure in order to infect the mulberry plants. Five to seven injection points were marked on each leaf, and the transiently transformed mulberry plants were grown in the dark for one day and then under normal conditions. Leaves from the same positions were collected at two, three, four, and five days post-treatment, as well as a control, and were immediately frozen in liquid nitrogen and stored at −80 °C for total RNA extraction and the determination of physiological and biochemical indicators.

### 4.10. Detection of Physiological and Biochemical Indicators

Samples of mulberry leaves were collected from all experimental groups at different time points (2 d, 3 d, 4 d, 5 d, and control) and subsequent analyses were conducted to determine the levels of proline (Pro), soluble protein, and malondialdehyde (MDA) within the leaves. Additionally, the enzyme activities of peroxidase (POD) and superoxide dismutase (SOD) were assessed. These analyses were performed using assay kits supplied by Suzhou Keming Biotechnology Co., Ltd., Suzhou, China. All testing procedures strictly adhered to the manufacturer’s instructions, and for each parameter, three biological replicates were used. Data were subsequently subjected to processing and analysis using GraphPad Prism 7 software.

### 4.11. Luciferase Reporter Assay

pmirGLO, pmirGLO-Maspl3-WT, or pmirGLO-Maspl3-MT was co-transfected with miR156x mimics or miR NC into 293T cells by Lipofectamine-mediated gene transfer. The relative luciferase activity was normalized to Renilla luciferase activity 48 h after transfection.

### 4.12. GST (Glutathione S-Transferase) Pull-Down Assays

A GST pull-down assay was performed by mixing 0.5 mg of GST-tagged fusion protein and total protein, incubating them on ice for 3 h, and then loading the mixture onto Glutathione Sepharose 4B resin columns. Following five washes with wash buffer, the proteins were eluted with wash buffer containing 15 mM reduced glutathione, and then separated using 12% SDS-PAGE. Finally, the proteins were transferred onto PVDF membranes (Millipore, Billerica, MA, USA) and probed with anti-GST (Sigma-Aldrich, Merck KGaA, Darmstadt, Germany). GST was used as negative controls. After the differential proteins were identified by silver staining, the target proteins were analyzed by mass spectrometry.

## 5. Conclusions

The differential ceRNA (competitive endogenous RNA) networks of dissected and whole mulberry leaves were obtained through ceRNA sequencing. Further analysis of the ceRNA network revealed that ceRNA regulation plays a significant role in leaf morphogenesis. The obtained ceRNA network was significantly enriched with differentially expressed genes, particularly those belonging to the *SPL* family. It is hypothesized that the miR156x/MaSPL3/MSTRG.25812.1 regulatory pathway is the primary driver of the differences in mulberry leaf shape. Pre-miR156x was successfully cloned and identified, and a miR156x dual overexpression vector, pCAMBIA-35S-GUS-miR156x, was constructed. Furthermore, a transient transformation system was established in mulberry trees. Transgenic mulberry trees exhibited alterations in gene expression levels and various physiological and biochemical parameters. This indicates that plant miRNAs can act as negative regulatory factors in gene expression to respond to changes in mulberry leaf morphology. The experimental results confirmed that the miR156x/*MaSPL3*/MSTRG.2582.1 regulatory pathway is indeed a potential factor underlying the differences in mulberry leaf shape.

The complete *MaSPL3* gene was cloned, and through multiple sequence alignments and phylogenetic tree analysis, a high degree of amino acid sequence homology was observed between the *MaSPL3* gene and other related species, indicating a close phylogenetic relationship with mulberry. The prokaryotic expression vector pET-20b (+)-*MaSPL3* was constructed and successfully induced for expression in *Escherichia coli*. The expression of the target protein was confirmed using methods such as SDS-PAGE and Western blot. Furthermore, a dual-luciferase reporter assay was conducted to provide experimental evidence of the targeted relationship between *MaSPL3* and miR156x. Subsequently, GST-Pull Down experiments were performed, leading to the identification of multiple proteins that interact with *MaSPL3*. These findings suggest that the miR156x/*MaSPL3*/MSTRG.25812.1 regulatory model is one of the primary factors contributing to differences in mulberry leaf.

## Figures and Tables

**Table 1:** Statistical table of differential miRNA–different target gene pairs.

Target_Type	Target_Num	miRNA_Num	Pairs_Num
mRNA	64	31	88
lncRNA	9	9	11
circRNA	4	4	6

**Table 2:** Summary of protein identification information statistics.

Sample	Total Number of Mass Spectra	Number of Identified Spectra	Spectral Interpretation Rate (%)	Number of Identified Peptide Segments	Number of Identified Proteins	Unique-2
SY	7096	915	12.89	115	48	26
DZ	2487	574	23.08	3	2	1

**Table 3:** Information about SY and DZ experimental group proteins.

Protein ID	Coverage (%)	Mass (Da)	Unique Peptide
Experimental group (SY)			
sp|C0Z361|CPNB3_ARATH	17.92	63,324.0	10
sp|P21238|CPNA1_ARATH	17.58	62,071.3	8
sp|P19366|ATPB_ARATH	16.67	53,933.4	5
sp|Q9LTX9|HSP7G_ARATH	9.47	76,995.9	6
sp|O65719|HSP7C_ARATH	10.17	71,147.0	7
sp|Q9LD57|PGKH1_ARATH	11.43	50,111.2	3
sp|P53496|ACT11_ARATH	14.59	41,674.3	4
sp|Q9C5A9|ATPBO_ARATH	12.16	59,858.6	4
sp|O03042|RBL_ARATH	6.89	52,954.7	3
tr|F4IGL5|F4IGL5_ARATH	9.51	41,807.3	3
Control group (DZ)			
sp|P59259|H4_ARATH	21.36	11,409.3	2
sp|P53497|ACT12_ARATH	4.77	41,794.5	1

## Data Availability

The original data sets described in the study are included in the article/Supplementary Materials. Further inquiries can be addressed to the corresponding author.

## Supplementary Materials

The following supporting information can be downloaded at: https://www.mdpi.com/article/10.3390/ijms242316860/s1.
