# Supplementary material for: The Competing Endogenous RNAs Regulatory Genes Network Mediates Leaf Shape Variation and Main Effector Gene Function in Mulberry Plant (Morus alba)
Source: Int J Mol Sci. 2023 Nov 28;24(23):16860. doi: 10.3390/ijms242316860 (PMC10706577; doi:10.3390/ijms242316860)
Supplement: Supplementary file 1 [file ijms-24-16860-s001.zip › Supplementary Materials.pdf]

Supplementary Materials：

Supplementary tables

Table S1. Statistics of the number of tested genes in each sample

sample	refer_genes	sequenced_refer_genes(%)	novel_genes	sequenced_novel_genes(%)	total_genes	sequenced_total_genes(%)
all	21,636	17,910 (82.78%)	643	643 (100.00%)	22,279	18,553 (83.28%)
CK-1	21,636	16,092 (74.38%)	643	590 (91.76%)	22,279	16,682 (74.88%)
CK-2	21,636	14,509 (67.06%)	643	511 (79.47%)	22,279	15,020 (67.42%)
CK-3	21,636	15,566 (71.94%)	643	561 (87.25%)	22,279	16,127 (72.39%)
HL-1	21,636	16,513 (76.32%)	643	595 (92.53%)	22,279	17,108 (76.79%)
HL-2	21,636	16,386 (75.73%)	643	604 (93.93%)	22,279	16,990 (76.26%)
HL-3	21,636	15,898 (73.48%)	643	577 (89.74%)	22,279	16,475 (73.95%)

Table S2. Statistics of the number and tag abundance of new miRNAs identified in each sample

sample	total_abundance	mirna_num	tags_unique	tags_abundance	ratio(%)
all	56,539,678	386	1072	260,765	0.46
CK-1	8,708,224	308	704	52,170	0.60
CK-2	8,741,329	275	588	45,565	0.52
CK-3	6,839,844	292	781	23,581	0.34
HL-1	7,640,415	340	757	31,073	0.41
HL-2	10,703,169	355	803	40,986	0.38
HL-3	13,906,697	342	794	67,390	0.48

Table S3. Statistics of the number of lncRNA detected in each sample

sample	Refer_ lncRA	Sequenced_Refer_ lncRNA(%)	Novel_ lncRNAs	sequenced_ Novel_lncRNAs(%)	Total_ lncRNAs	sequenced_ Total_lncRNAs(%)
all	951	298 (31.34%)	8,726	8,725 (99.99%)	9,677	9,023 (93.24%)
CK-1	951	180 (18.93%)	8,726	6,617 (75.83%)	9,677	6,797 (70.24%)
CK-2	951	140 (14.72%)	8,726	5,685 (65.15%)	9,677	5,825 (60.19%)
CK-3	951	156 (16.40%)	8,726	6,037 (69.18%)	9,677	6,193 (64.00%)
HL-1	951	186 (19.56%)	8,726	6,807 (78.01%)	9,677	6,993 (72.26%)
HL-2	951	206 (21.66%)	8,726	7,194 (82.44%)	9,677	7,400 (76.47%)

Table S4. Statistics of the circRNA sequencing reads detected in each sample

Sample	Total	Unmapped(%)	Unique_Mapped(%)	Multiple_Mapped(%)	Total_Mapped(%)
CK-1	56,516,478	22,319,563 (39.49%)	16,118,634 (28.52%)	18,078,281 (31.99%)	34,196,915 (60.51%)
CK-2	44,218,836	17,466,225 (39.50%)	11,878,307 (26.86%)	14,874,304 (33.64%)	26,752,611 (60.50%)
CK-3	78,077,398	46,984,498 (60.18%)	14,322,807 (18.34%)	16,770,093 (21.48%)	31,092,900 (39.82%)
HL-1	72,300,802	29,570,466 (40.90%)	20,915,227 (28.93%)	21,815,109 (30.17%)	42,730,336 (59.10%)
HL-2	65,543,296	27,629,596 (42.15%)	19,278,987 (29.41%)	18,634,713 (28.43%)	37,913,700 (57.85%)
HL-3	76,172,430	32,365,582 (42.49%)	20,433,765 (26.83%)	23,373,083 (30.68%)	43,806,848 (57.51%)

Table S5. Differential negative correlation targeted miRNA-mRNA

miRNA	CK-1	CK-2	CK-3	HL-1	HL-2	HL-3	mRNA	CK-1	CK-2	CK-3	HL-1	HL-2	HL-3	rho	Symbol
**miR156-x**	**428.7195**	**360.6911**	**5821.0404**	**220.4170**	**178.4761**	**180.9024**	**ncbi_21398587**	**3.08**	**0.11**	**0.00**	**13.36**	**9.25**	**8.35**	**-0.771428571428571**	**SPL3**
miR156-x	428.7195	360.6911	5821.0404	220.4170	178.4761	180.9024	ncbi_21404628	0.29	0.00	0.06	1.38	1.73	4.22	-0.771428571428571	SPL7
miR156-x	428.7195	360.6911	5821.0404	220.4170	178.4761	180.9024	ncbi_21411327	0.00	0.00	0.00	0.15	0.28	0.31	-0.880406274042429	SPL6
miR157-x	263.4221	224.4234	5461.6489	132.8302	99.5044	64.2400	ncbi_21398587	3.08	0.11	0.00	13.36	9.25	8.35	-0.714285714285714	SPL3
miR157-x	263.4221	224.4234	5461.6489	132.8302	99.5044	64.2400	ncbi_21401011	1.87	0.96	1.48	5.01	4.97	5.54	-0.771428571428571	SPL14
miR157-x	263.4221	224.4234	5461.6489	132.8302	99.5044	64.2400	ncbi_21404628	0.29	0.00	0.06	1.38	1.73	4.22	-0.828571428571429	SPL7
miR157-x	263.4221	224.4234	5461.6489	132.8302	99.5044	64.2400	ncbi_21411327	0.00	0.00	0.00	0.15	0.28	0.31	-0.94112394811432	SPL6
miR2916-z	4.2806	6.5743	6.2913	11.6009	23.6915	49.0891	ncbi_21401843	1.48	0.42	1.79	0.57	0.57	0.13	-0.753702346348183	GOLS2
miR2916-z	4.2806	6.5743	6.2913	11.6009	23.6915	49.0891	ncbi_21403045	78.96	72.65	135.21	20.22	38.48	22.62	-0.771428571428571	NIA
miR399-x	0.9878	1.4942	6.2913	0.01	0.01	0.6060	ncbi_21390492	0.13	0.00	0.06	1.52	1.33	1.27	-0.927633657043918	ANT
miR399-x	0.9878	1.4942	6.2913	0.01	0.01	0.6060	ncbi_21390620	0.39	0.39	0.03	1.97	1.72	3.50	-0.794117647058823	--
miR399-x	0.9878	1.4942	6.2913	0.01	0.01	0.6060	ncbi_21395176	0.73	0.33	0.26	3.13	3.36	3.46	-0.811679449913428	rhp16
miR399-x	0.9878	1.4942	6.2913	0.01	0.01	0.6060	ncbi_21408525	28.61	10.12	18.25	55.04	51.31	47.20	-0.927633657043918	FBA3
miR408-y	230.1651	170.0358	228.0602	56.8444	62.1244	85.7544	ncbi_21406932	10.58	0.94	0.45	20.89	26.53	19.79	-0.771428571428571	ARPN
miR482-y	29478.2497	59255.5360	37554.4395	24297.4933	20012.4881	16765.7438	ncbi_112094938	0.00	0.00	0.00	0.08	0.08	0.28	-0.925820099772551	RPPL1
miR529-x	7.5734	5.3790	40.1071	0.5800	1.0530	5.1513	ncbi_112090714	25.29	8.13	5.89	39.07	44.80	34.24	-0.885714285714286	MORF5
miR529-x	7.5734	5.3790	40.1071	0.5800	1.0530	5.1513	ncbi_21388550	30.04	9.13	10.60	57.76	57.36	55.42	-0.828571428571429	nusB
miR529-x	7.5734	5.3790	40.1071	0.5800	1.0530	5.1513	ncbi_21390050	11.57	2.55	4.29	21.06	25.89	19.79	-0.771428571428571	RPL15
miR529-x	7.5734	5.3790	40.1071	0.5800	1.0530	5.1513	ncbi_21390254	49.48	20.00	36.68	112.47	105.80	80.60	-0.828571428571429	RPL21

Table S6. The reverse transcription and RT-qPCR primer sequences for 12 mRNAs expression analysis

Gene	Primer	Sequence (5'-3')
B-actin	forward primer	CCGTTCTCTCCCTTTACGCC
	reverse primer	AGACGGAGAATAGCATGGGGA
ncbi_21398587	forward primer	CTGCCCGAGTTCAACAACCT
	reverse primer	GCTGAGGATGACTTGGCGTT
ncbi_21404628	forward primer	GAACATCGAGCCCCACCATC
	reverse primer	CTAGCAGGGACAAACCCGAA
ncbi_21401011	forward primer	AAGTCATTGTCGCCGGTCTT
	reverse primer	GCTTCCTCCGACGCTCATT
ncbi_21401843	forward primer	GAATTGGAGAGACAGCCCACA
	reverse primer	CCGGCGTTGAAGTAGAGAGG
ncbi_21403045	forward primer	TTAGGGTTTTCCGCTTCGCT
	reverse primer	TCGTCAATGGAAGTCGTGGG
ncbi_112091005	forward primer	AGCTGAAGAAATGGGGCAGAA
	reverse primer	TGGGCCGAACACTCATACTC
ncbi_21400106	forward primer	TGCAGAAAAACTTGGGCGGA
	reverse primer	CTCTTCACCAAACACCCGAGA

ncbi_21390050	forward primer	GCAAGAGGCCGGTTCCAAAG
	reverse primer	GACCACCCAGCTTACGTCC
ncbi_21390254	forward primer	TTGTTTTTCCTGGGCGGTTT
	reverse primer	GCTTGCCAATGTATGTGCTGT
ncbi_21390777	forward primer	CCGTGCTCAAGTGTTGCTTC
	reverse primer	TTCAAATCCCCCTCCGCAAA
ncbi_21395231	forward primer	TTCCCCGAATGGGTTTCCAC
	reverse primer	CTCCACGGCCTCCGAAATAG
ncbi_21389036	forward primer	GTCCTCTCTTGCGACGGCTT
	reverse primer	ATCGGAGGTTAGGCTTGTGAG

Table S7. The stem-loop reverse transcription and RT-qPCR primer sequences for 10 miRNAs expression analysis

Gene	Primer	Sequence (5’-3’)
U6	forward primer	GGGGACATCCGATAA
	reverse primer	ATTTGGACCATTTCTC
universal	reverse primer	CCAGTGCAGGGTCCGAGGTAT
miR529-x	stem-loop RT primer	GTCGTATCCAGTGCAGGGTCCGAGG TATTCGCACTGGATACGACGCTGTA
	forward primer	GCGCGCGAAGAGAGAGAG
miR156-x	stem-loop RT primer	GTCGTATCCAGTGCAGGGTCCGAGG TATTCGCACTGGATACGACGTGCTC
	forward primer	GCGCGCTTGACAGAAGATAGA
miR2916-z	stem-loop RT primer	GTCGTATCCAGTGCAGGGTCCGAGG TATTCGCACTGGATACGACTGATCG
	forward primer	CCACACTGGGGCTCGAAGA
miR319-y	stem-loop RT primer	GTCGTATCCAGTGCAGGGTCCGAGG TATTCGCACTGGATACGACGGGAGC
	forward primer	GAGCGCTTGGACTGAAGGGA
miR399-x	stem-loop RT primer	GTCGTATCCAGTGCAGGGTCCGAGGT ATTCGCACTGGATACGACGCCAAA

	forward primer	GCCCGGAGGGCTTCTCTCT
novel-m0038-3p	stem-loop RT primer	GTCGTATCCAGTGCAGGGTCCGAGG TATTCGCACTGGATACGACAGGAGC
	forward primer	GCCCGGGAAGTTTGGTATT
miR156-y	stem-loop RT primer	GTCGTATCCAGTGCAGGGTCCGAGG TATTCGCACTGGATACGACGGATGA
	forward primer	CTTCCCGCTCTCTATGCTTCTG
miR166-x	stem-loop RT primer	GTCGTATCCAGTGCAGGGTCCGAGG TATTCGCACTGGATACGACCCTCGA
	forward primer	CAACACGGAATGTTGTCTGGC
miR168-y	stem-loop RT primer	GTCGTATCCAGTGCAGGGTCCGAGG TATTCGCACTGGATACGACATTCAG
	forward primer	AATTAACCCGCCTTGCATCAA
miR482-z	stem-loop RT primer	GTCGTATCCAGTGCAGGGTCCGAGG TATTCGCACTGGATACGACGGTATG
	forward primer	TAACGATTTTCCCAACACCACC

Table S8. The reverse transcription and RT-qPCR primer sequences for 9 lncRNAs expression analysis

Gene	Primer	Sequence (5’-3’)
β- actin	forward primer	AAGAGCGGTTCCTCGGTTG
	reverse primer	TGGTTGGAAGAGGACTTGTGG
MSTRG.25812.1	forward primer	TCCCGGTAGCTCTAT
	reverse primer	GTCCGACATGGTTGA
MSTRG.2225.1	forward primer	AGCGTTCCAACTCCA
	reverse primer	ATACACTCGCCACCC
MSTRG.7548.1	forward primer	AATGAGTGCCGACCTT
	reverse primer	TTTGTAGTGTAGCCTTGT
MSTRG.14005.1	forward primer	GCTCCTAGCCCTTCT
	reverse primer	CTTCAACGACCTTATTT
MSTRG.22209.1	forward primer	TGACCAGTTGGGAATA
	reverse primer	TGTTTGATCGCCTTG

MSTRG.20836.1	forward primer	TAGTCAAAGGAGGGA
	reverse primer	TAAAGGAATGGTCGT
MSTRG.8298.1	forward primer	CTCGTCCTTTCCAAC
	reverse primer	TCTCAGGTCCAGTTTC
MSTRG.13866.1	forward primer	GAGAATACGGAAGCAA
	reverse primer	CAGCCTGACTCCAAAC
MSTRG.25454.1	forward primer	TTCGCTCTTGGTGGTC
	reverse primer	CTGCGTTGCGGTTAT

Table S9. The reverse transcription and RT-qPCR primer sequences for 10 circRNAs expression analysis

Gene	Primer	Sequence (5'-3')
18S rRNA	forward primer	GCAAGACCGAAACTCAAAG
	reverse primer	TGTCAAGGGCTGGTAAGG
novel_circ_000060	forward primer	TGATGTTGGTGGTCATAC
	reverse primer	AGACAGCCTTATAGTTTTG
novel_circ_000346	forward primer	ATTTCTCGGAAGTTGCTC
	reverse primer	TTCCCAGTTTATTTTGTCTA
novel_circ_000071	forward primer	TCAATGGACTTCCCAAAT
	reverse primer	AGACACCACGGGTACAGC
novel_circ_000098	forward primer	TCAATGGACTTCCCAAAT
	reverse primer	AGACACCACGGGTACAGC
novel_circ_000123	forward primer	TGGATTTGGGCAAGGAAG
	reverse primer	CCACCGCCACCAACACTA
novel_circ_000147	forward primer	GATGGCTTATGGACATTC
	reverse primer	TTTACAAAGGCTTGGAGA
novel_circ_000214	forward primer	CCGAGTGAGGAGACAAAG
	reverse primer	CGGAGAAAGGAGCAGGAT
novel_circ_000240	forward primer	ATGGGAGGTGGAGATGGTG
	reverse primer	CTCGGATTCGCAACTGGA
novel_circ_000245	forward primer	CTGTTGAAGCTGCCTTTG
	reverse primer	GCTATTGACAACCTGCTAT

novel_circ_000254	forward primer	TCGGATGGAAGAACGGGTGA
	reverse primer	GGCCATGTTCGGCACAACT

Table S10. Primers used for cloning

Gene name	Primers	Sequence information (5’-3’)
*MaSPL*3	F1	ATGATGGAGTGGAATTCAAAAG
	R1	TATTCCCATCACTTCAAGTAG
pET-20b（+）-*MaSPL*3	F2	gccatATGATGGAGTGGAATTCAAAAG
	R2	ccctcgagggTATTCCCATCACTTCAAGTAG

Supplementary Figures

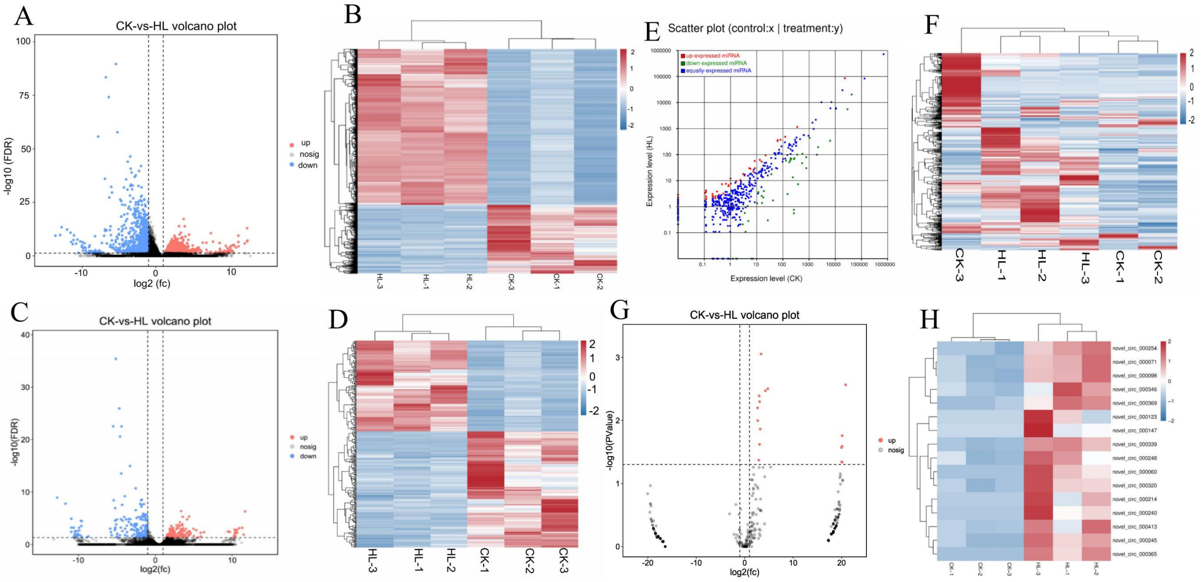

Figure S1. Volcan plot and heat maps showing the distribution pattern of the DEGs of the ceRNAs. (A, B) mRNAs, (C, D) lncRNAs, (E, F) miRNAs, (G, H) circRNAs.

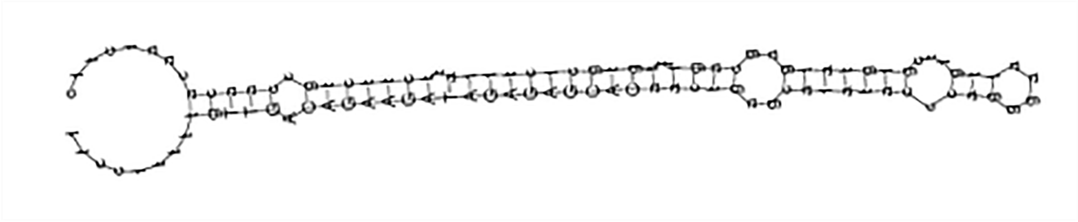

Figure S2. The secondary structure of pre-miR156x in mulberry

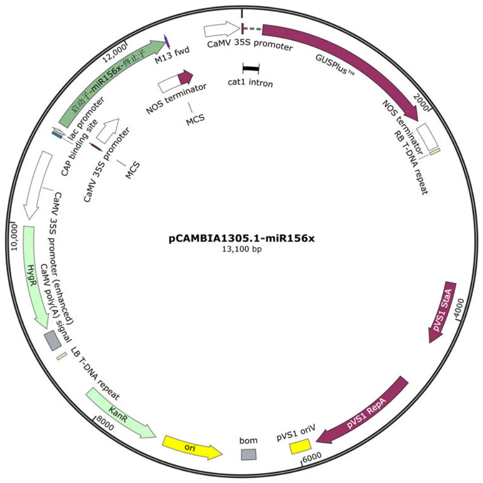

Figure S3. Construction diagram of mulberry dual-expression recombinant vector

```
                10        20        30        40        50        60
1       ATGGAGTGGAATTCAAAAGCTTCCTCATTATGGGACTGGGAGAACCTTTTTGAGCTTGAG
1        M  E  W  N  S  K  A  S  S  L  W  D  W  E  N  L  F  E  L  E
                70        80        90       100       110       120
61      GCGAAAGCAACCGAGAATCCTAAGAAGCTACTACAACGAATGGATTGCTTTGTTGAAGTA
21       A  K  A  T  E  N  P  K  K  L  L  Q  R  M  D  C  F  V  E  V
               130       140       150       160       170       180
121     GATCGAGGAATTAGCTCCGGATCTCTCCATTCACCTGGGGATGGTGGGGGTAGCAGCGCA
41       D  R  G  I  S  S  G  S  L  H  S  P  G  D  G  G  G  S  S  A
               190       200       210       220       230       240
181     GGAACCGGTGGTTCAGGTTCCGATTTGGGTCATGCTTCATCGAAGAGCTCCATATCGGCT
61       G  T  G  G  S  G  S  D  L  G  H  A  S  S  K  S  S  I  S  A
               250       260       270       280       290       300
241     TCCATCAATTCTTCATCAATTGGGGAAAGCAAGACATCCAACAAATTTACTCTCGACGGT
81       S  I  N  S  S  S  I  G  E  S  K  T  S  N  K  F  T  L  D  G
               310       320       330       340       350       360
301     TTCGAACCTTTTCCAAAGGATTCAAGGAGTGAAAATGAGTCTGCCCGAGTTCAACAACCT
101      F  E  P  F  P  K  D  S  R  S  E  N  E  S  A  R  V  Q  Q  P
               370       380       390       400       410       420
361     AGATCTTCACCAACACCTGAAATTTCAGCTGGCTCTGGTGAGCCATTGCTCAGTTTAAAG
121      R  S  S  P  T  P  E  I  S  A  G  S  G  E  P  L  L  S  L  K
               430       440       450       460       470       480
421     TTGGGCAAGCGGATGTACTTCAAAGATATTTGTTCAGAAAACAACGCCAAGTCATCCTCA
141      L  G  K  R  M  Y  F  K  D  I  C  S  E  N  N  A  K  S  S  S
               490       500       510       520       530       540
481     GCCCCGTCTGTAGCACCCATGTCATCTGCTACAACGGCTAAGAGAAGCAAGTCCACTTGC
161      A  P  S  V  A  P  M  S  S  A  T  T  A  K  R  S  K  S  T  C
               550       560       570       580       590       600
541     CAGAGTAATTTCATGCCTCGCTGCCAAGTTGAAGGCTGCAACCTTGATCTTTCCTCGGCA
181      Q  S  N  F  M  P  R  C  Q  V  E  G  C  N  L  D  L  S  S  A
               610       620       630       640       650       660
601     AAAGATTACCATCGAAAGCATAGAATTTGTGAAAGTCATTCCAAATCTCCAAGGGTCATT
201      K  D  Y  H  R  K  H  R  I  C  E  S  H  S  K  S  P  R  V  I
               670       680       690       700       710       720
661     GTAGGCGGTGTGGAGCGTCGGTTTTGCCAGCAGTGTAGCAGGTTCCACGGTCTGTCTGAA
221      V  G  G  V  E  R  R  F  C  Q  Q  C  S  R  F  H  G  L  S  E
               730       740       750       760       770       780
721     TTTGATGAAAAGAAGAGAAGCTGTCGAAGACGTCTTTCAGATCACAACGCAAGGCGCCGC
241      F  D  E  K  K  R  S  C  R  R  R  L  S  D  H  N  A  R  R  R
               790       800       810       820       830       840
781     AAGCCACAGCCTGAAGCCGTCCGGTTAAATCCAGCGAGACTTTCTTCACTATATGACGAG
261      K  P  Q  P  E  A  V  R  L  N  P  A  R  L  S  S  L  Y  D  E
               850       860       870       880       890       900
841     ACACAGAAGATGAGCCTTGTGTTTGACCAAGCCCCGCGCCTTTATACAAGGCATGCTGCA
281      T  Q  K  M  S  L  V  F  D  Q  A  P  R  L  Y  T  R  H  A  A
               910       920       930       940       950       960
901     AATTTTACATGGGATCATAGCACTGATAGCTCTAAGTTGGCGCATACAAGAGAGTACTTG
301      N  F  T  W  D  H  S  T  D  S  S  K  L  A  H  T  R  E  Y  L
               970       980       990      1000      1010      1020
961     TCAAGGCCTGCGAAAACAGGAGCCGCACCTGGACAGATTTTTTTGCCCAACAATGAAATG
321      S  R  P  A  K  T  G  A  A  P  G  Q  I  F  L  P  N  N  E  M
              1030      1040      1050      1060      1070      1080
1021    CCTAGCAGTATTTCCATGCTTTATCCTCAGGATTCCGGTAGACTAACGCCAAATAAAAAC
341      P  S  S  I  S  M  L  Y  P  Q  D  S  G  R  L  T  P  N  K  N
              1090      1100      1110      1120      1130      1140
1081    ACTGCCCCTGAGGTTCTCCACTCAGGGTTAGAAGAATCTGTGGTGTCGACAGAGCTAAAT
361      T  A  P  E  V  L  H  S  G  L  E  E  S  V  V  S  T  E  L  N
              1150      1160      1170      1180      1190      1200
1141    GCGACACAGGATCTTCATCGTGCTCTCTCTCTTCTGTCAACTAGTTCATGGTGTTCGCGC
381      A  T  Q  D  L  H  R  A  L  S  L  L  S  T  S  S  W  C  S  R
              1210      1220      1230      1240      1250      1260
1201    GAAACAAAGATTGTTTCTCTAGATACTTGTTCTACCGCCCTCAACAACACAAATCACCAT
401      E  T  K  I  V  S  L  D  T  C  S  T  A  L  N  N  T  N  H  H
              1270      1280      1290      1300      1310      1320
1261    AGCAGCTGCAGCAACGGCATGGTTCAACCCGCGATGCATCAAATGACCACCACACAGACT
421      S  S  C  S  N  G  M  V  Q  P  A  M  H  Q  M  T  T  T  Q  T
              1330      1340      1350      1360      1370      1380
1321    CAAGGCTTTCCTTTTGCTTCAGAGTTCTGGCAGACCCAACAGCCAACAGCTATTGATGCG
441      Q  G  F  P  F  A  S  E  F  W  Q  T  Q  Q  P  T  A  I  D  A
              1390      1400      1410      1420      1430      1440
1381    GCATTTCCACCTACCCCCCACACACGCAGTAATAGTAATGCCAATGCCACCACCCACTTT
461      A  F  P  P  T  P  H  T  R  S  N  S  N  A  N  A  T  T  H  F
              1450      1460      1470      1480      1490      1500
1441    CAAGATCTCCATTTGATCAAACCTCCCGGTGACACTGCTTTTTATTCCCATCACTTCAAG
481      Q  D  L  H  L  I  K  P  P  G  D  T  A  F  Y  S  H  H  F  K
1501    TAG
501      *
```

Figure S4. The nucleotide and deduced amino acids sequence of mulberry MaSPL3

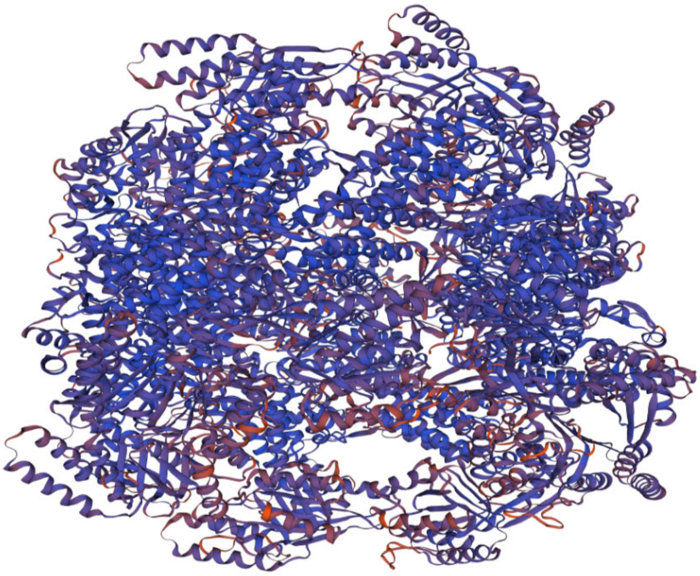

Figure S5. The protein tertiary structure of mulberry *MaSPL*3

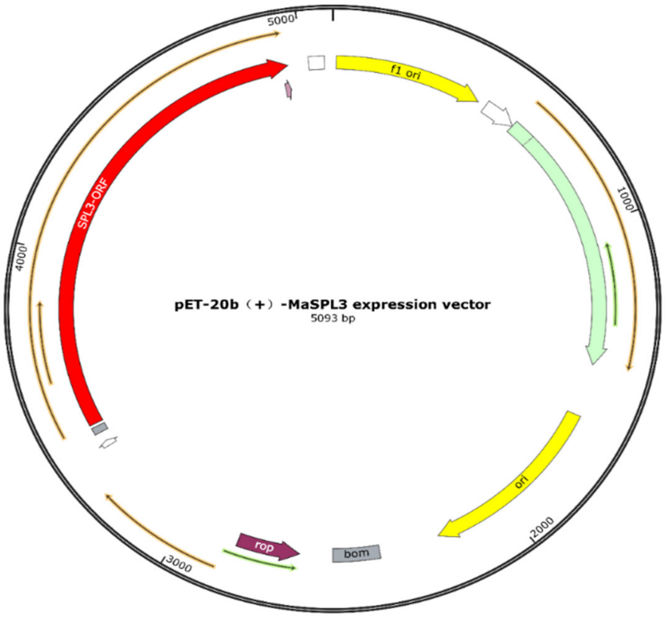

Figure S6. Construction diagram of prokaryotic expression vector pET-20b (+) - *MaSPL*3 of mulberry
